# The European Space Agency’s Comet Interceptor lies in wait

**DOI:** 10.1038/s41467-019-13470-1

**Published:** 2019-11-28

**Authors:** Colin Snodgrass, Geraint H Jones

**Affiliations:** 1 Institute for Astronomy, University of Edinburgh, Royal Observatory, Edinburgh, EH9 3HJ UK; 2 Mullard Space Science Laboratory, University College London, Holmbury St. Mary, Dorking, Surrey, RH5 6NT UK

**Keywords:** Astronomy and planetary science, Astronomy and astrophysics, Asteroids, comets and Kuiper belt

## Abstract

The European Space Agency (ESA) recently selected *Comet Interceptor* as its first ‘fast’ (F-class) mission. It will be developed rapidly to share a launch with another mission and is unique, as it will wait in space for a yet-to-be-discovered comet.

## A new comet mission

European Space Agency (ESA) has a remarkable history of successful missions to explore comets, despite the many challenges associated with visiting these unpredictable and active bodies on highly eccentric orbits. They are high value targets for scientific exploration, as they preserve, to a greater or lesser degree, the ices formed at the birth of our Solar System. The *Giotto* mission was ESA’s first deep-space mission^1^. It returned the most detailed images of the nucleus of comet 1P/Halley^2^, as part of the armada of spacecraft that encountered it in 1986. Although *Giotto* was damaged by dust grain impacts during its high-speed (68 km s^−1^) close (600 km) encounter, it returned data that set the framework for our understanding of comets ever since: proving the nucleus was small and dark, with ‘jets’ of activity from discrete areas. Three decades later, ESA again excelled itself with the *Rosetta* mission to comet 67P/Churyumov-Gerasimenko; the first spacecraft to orbit a comet, providing an unprecedented two year investigation of its evolution as it approached and receded from the Sun, and the first soft landing on a comet nucleus, delivering the *Philae* lander to perform experiments on the surface^3^. *Rosetta* has led to a revolution in cometary science, and the processes of planet formation, with more than 1000 papers already published; some of the key results are summarised in early review articles [e.g. refs. ^4–6^].

Work on the rich dataset from this mission will continue for many years, but already it has raised new questions that will require a future mission to answer. Some of these, such as how the smallest building blocks were originally assembled, and where the constituent particles formed, will require an even more ambitious (and large) future mission to return a sample of a comet for study in laboratories on Earth. The comet science community is already planning such a mission, but it is many decades away^7^. One of the surprising results from *Rosetta* was the extent to which the nucleus surface had been altered, by erosion or fall back of material, as a result of 67P’s repeated close perihelion passages^8^. This motivates study of more pristine comets that have not undergone multiple cycles of activity. *Comet Interceptor* will be the first mission to visit a Dynamically New Comet (DNC), i.e. one entering the inner Solar System for the first time since it formed, and only beginning to experience activity due to being close enough to the Sun to raise the temperature above the sublimation point of its constituent ices.

DNCs are a subset of the long period comets that come from the Oort cloud, at the very edge of our Solar System, reaching half-way to the next star. Most comet missions to date have visited short period comets, like 67P, which come from the less distant Kuiper Belt region, and have experienced heating on many orbits near to the Sun. Oort cloud objects were scattered to this distant reservoir during the formation and early evolution of our Solar System, and have been preserved there ever since. They are therefore some of the most pristine ‘building blocks’ from the era of planet formation, and, when finally scattered back into the inner Solar System to feel the warmth of the Sun, produce some of the most spectacular bright comets. The high activity levels, and therefore brightness, of such comets means that they have been popular telescopic targets over the years, but are difficult to explore with spacecraft. However, the primary reason they have yet to be visited is that their appearance is fleeting—they are typically discovered only a few months to a year before they pass perihelion, before returning to the distant outer Solar System, with orbital periods of tens of thousands of years, which mean they will be seen only once by humanity. This has always been incompatible with the timescales to design, build and launch a space mission, which can take decades.

## ESA’s new fast approach

**Fig. 1:**
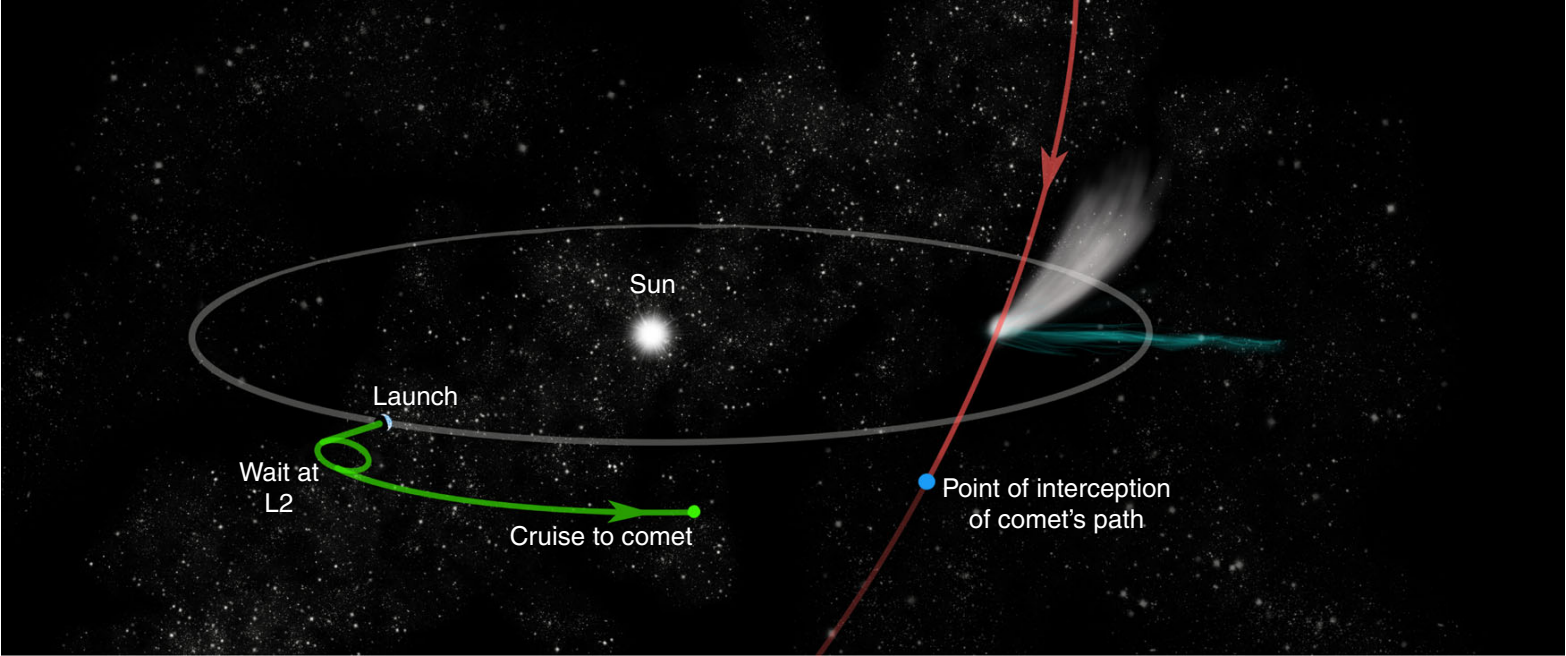
Sketch of mission phases. The spacecraft launches from Earth and waits at L2 until a suitable comet is discovered, before departing on an interplanetary cruise to be in the right place at the right time to intercept the comet as it crosses the ecliptic plane. The smaller probes are released just before arriving at the comet. The trajectory of the spacecraft is in green, while the comet’s orbit is in red.

*Comet Interceptor* will be developed on a relatively rapid timescale for ESA, to take advantage of extra space available on the launcher of the medium (M-class) mission *Ariel*, which is due to launch in 2028^9^. Such a timescale is still not fast enough to react to any comet we currently know, but the specific opportunity offered by the F-class call, i.e. a free launch to a halo orbit around the Sun-Earth L2 Lagrangian point, enables an unique approach: to design and launch a mission without a known target, that can wait in space for a suitable comet to be discovered. The L2 point is where the gravitational pull of the Sun and Earth balance to produce a stable point in space 1.5 million km away from Earth, on the planet’s night side. It is the favoured parking location for space observatories, as an orbit can be maintained there with little fuel, while keeping a constant distance from Earth and stable illumination. L2 is also the ideal parking location for *Comet Interceptor*, as both station keeping there and departure for the comet, once it is identified, take relatively little fuel (Fig. 1).

**Fig. 2:**
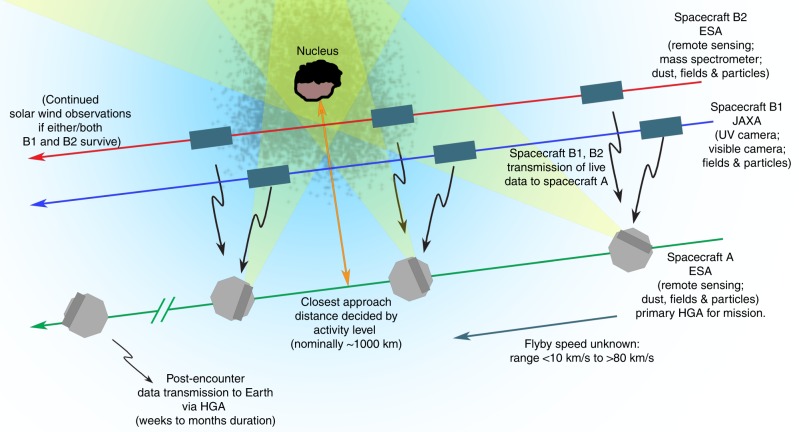
The proposed general encounter sequence for Comet Interceptor, not to scale. Green, blue and red lines are the trajectories of spacecraft (SC) A, B1 and B2, respectively. SC A passes farthest from the nucleus, performing remote sensing observations and receiving data from SC B1 and B2, which follow near-parallel trajectories nearer the nucleus. Post-encounter, SC A transmits stored data from all three platforms to Earth. The final SC, payload and precise flyby scenario may change as the mission development proceeds.

The F-class call also encouraged proposals that would employ a multiple spacecraft architecture, i.e., a small ‘swarm’ of similar craft, or a ‘mothership’ that releases smaller probes. *Comet Interceptor* follows the latter approach, which is also ideal for encountering a DNC: multiple spacecraft mean that the comet will be seen from different angles during the fly-by, building up a 3D picture of the nucleus, coma, and interaction with the solar wind. This will allow differences in time (e.g. due to the changing activity of the comet) and in space (e.g. due to inhomogeneities in the outgassing pattern) to be separated, which has not been possible in any previous fly-by mission, or even with *Rosetta*, which could only sample one area of the coma at a time. In addition, the mothership and probes architecture of *Comet Interceptor* solves one of the difficulties in visiting a DNC: the relatively high and unpredictable activity levels. The dust grains in the coma present a significant hazard as the spacecraft pass through at 10–80 km s^−1^. *Comet Interceptor* will be made up of a mothership (spacecraft A, Fig. 2) that will perform a relatively distant fly-by, at around 1000 km from the nucleus, where the dust flux will be manageable and the safety of the spacecraft can be assured, and two smaller probes (spacecraft B1 and B2), which will make closer approaches to perform high-risk/high-return measurements, but may not survive the whole encounter. This is a radically different approach to mission design, and the usual highly conservative approach of spacecraft engineering, that enables scientific measurements that could not previously be attempted. The approach builds on the many recent advances in small spacecraft technology, both in developments for easier access to Earth orbit in the ‘new space’ industry, and experience in deep space exploration, especially with the highly successful Japanese *Hayabusa 2* mission to asteroid 162173 Ryugu^10^. One of *Comet Interceptor*’s small probes, B1, will be supplied by the Japan Aerospace Exploration Agency (JAXA), and will build directly on heritage from *Hayabusa 2* (spacecraft A and B2 will be built by ESA).

## Intercepting an unknown target

The most unique aspect of *Comet Interceptor* is the fact that it must be designed without knowing its target, or the precise geometry of the fly-by encounter. The project is possible because large sky surveys are now finding incoming comets with greater warning times, of a few years at least, and the Large Synoptic Survey Telescope (LSST), currently under construction in Chile^11^, is expected to greatly increase our ability to discover comets years before they reach perihelion. Simulations of LSST performance, based on the best current understanding of the underlying population of Oort cloud comets from the Pan-STARRS survey^12^, suggest that ~5 years between discovery and interception is likely, and the target comet may be found before the mission is launched, but it still will not be known before the design must be fixed. This means that the mission must be designed to be as flexible as possible, and able to cope with a wide range of targets and encounter geometries.

Such an approach naturally enables a great deal of choice later in the project, and leaves open the exciting possibility of encountering an interstellar object (ISO) like 1I/‘Oumuamua. *Comet Interceptor* would be well equipped to characterise an ISO, as all indications from 1I/‘Oumuamua suggest that it is a comet-like body, albeit with a very low dust production^13^. However, the probability of a reachable ISO being discovered during the right period appears to be very low—the lack of observable dust coma around 1I/‘Oumuamua means that any similar body would not be discovered until very shortly before its closest approach, even with LSST. The recent discovery of the second ISO, 2I/Borisov, gives slightly more reason to be hopeful—this object looks like a typical comet^14^, and was discovered inbound.

In any case, *Comet Interceptor* will demonstrate the possibility of this approach, opening the possibility of a dedicated ISO mission to be proposed later in the coming decade, once LSST has provided better statistics on the true arrival rate of such objects. Such a mission would probably need to be of a larger class, with more mass allocated to fuel to enable a rapid response over a wider possible intercept range, but presents a scientifically compelling case—the only way to study material from another stellar system in situ. Additionally, *Comet Interceptor* will be a proof of concept for the use of a similar ‘rapid reaction’ spacecraft waiting in orbit for the discovery of close-approaching Near Earth Asteroids, which have been proposed for both planetary defence and commercial resource utilisation approaches.

More information about the mission, and updates on its development, can be found at Comet Interceptor Mission home: http://www.cometinterceptor.space.
